# Metabolomic Alterations in Patients with Obesity and the Impact of Metabolic Bariatric Surgery: Insights for Future Research

**DOI:** 10.3390/metabo15070434

**Published:** 2025-06-26

**Authors:** Ioanna A. Anastasiou, Dimitris Kounatidis, Miikka-Juhani Honka, Natalia G. Vallianou, Eleni Rebelos, Nikolaos Nektarios Karamanolis, Maria Dalamaga, Constantinos Pantos, Iordanis Mourouzis

**Affiliations:** 1 Diabetes Center, First Department of Propaedeutic Internal Medicine, Medical School, National and Kapodistrian University of Athens, Laiko General Hospital, 115 27 Athens, Greece; anastasiouiwanna@gmail.com (I.A.A.); elenirebelos@gmail.com (E.R.); 2 Department of Pharmacology, National and Kapodistrian University of Athens, 115 27 Athens, Greece; cpantos@med.uoa.gr (C.P.); imour@med.uoa.gr (I.M.); 3 Turku PET Centre, University of Turku, 20014 Turku, Finland; mjhonk@utu.fi; 4 Institute of Clinical Physiology, National Research Council (CNR), 56100 Pisa, Italy; 5 First Department of Internal Medicine, Sismanogleio General Hospital, 151 26 Athens, Greece; natalia.vallianou@gmail.com; 6 Department of Clinical and Experimental Medicine, University of Pisa, 56126 Pisa, Italy; 7 Second Department of Internal Medicine, Medical School, National and Kapodistrian University of Athens, Hippokration General Hospital, 115 27 Athens, Greece; inektkaramanolis@gmail.com; 8 Department of Biological Chemistry, National and Kapodistrian University of Athens, 115 27 Athens, Greece; madalamaga@med.uoa.gr

**Keywords:** amino acids, bile acids, biomarkers, endocannabinoids, lipid derivatives, metabolic bariatric surgery, metabolomics, obesity, Roux-en-Y gastric bypass, type 2 diabetes

## Abstract

Metabolomics has emerged as a vital tool for understanding the body’s responses to therapeutic interventions. Metabolic bariatric surgery (MBS) is widely recognized as the most effective treatment modality for severe obesity and its associated comorbidities. This review seeks to analyze the current evidence on the metabolomic profiles of patients with obesity and the impact of various bariatric surgical procedures, with the objective of predicting clinical outcomes, including weight loss and remission of type 2 diabetes (T2D). The data gathered from original studies examining metabolomic changes following MBS have been meticulously compiled and summarized. The findings revealed significant alterations in metabolites across various classes, including amino acids, lipids, energy-related compounds, and substances derived from the gut microbiota. Notably, elevated preoperative levels of specific lipids, such as phospholipids, long-chain fatty acids, and bile acids, were correlated with postoperative remission of T2D. In conclusion, metabolite profiling holds great promise for predicting long-term responses to different bariatric surgery procedures. This innovative approach has the potential to facilitate personalized treatment strategies and optimize the allocation of healthcare resources.

## 1. Introduction

The rising global prevalence of obesity represents a critical public health concern [1]. Obesity constitutes a major risk factor for numerous medical conditions, particularly non-communicable diseases [2,3]. Metabolic bariatric surgery (MBS) has emerged as an effective therapeutic intervention for individuals with severe obesity who fail to achieve adequate clinical outcomes through lifestyle modifications and conventional pharmacotherapy. This is particularly important given that weight loss has been consistently associated with improvements in various obesity-related conditions, including type 2 diabetes (T2D) [2,3]. Beyond promoting sustained weight reduction, MBS elicits a wide array of metabolic benefits, justifying its classification as a “metabolic” surgical intervention. These benefits include reduced incidence and mortality related to cardiovascular disease (CVD) [4,5,6,7,8,9,10], improvements in metabolic-dysfunction-associated steatotic liver disease (MASLD) [11,12], positive effects on polycystic ovary syndrome (PCOS) [13], and improved renal function [14].

Despite these favorable clinical outcomes, the precise mechanisms underlying the metabolic improvements observed postoperatively remain incompletely understood [15,16]. Clarifying these mechanisms is essential for advancing the understanding of the role of the foregut in systemic metabolism, particularly with respect to glucose and energy regulation. This knowledge may contribute to the development of novel therapeutic strategies for obesity and its comorbidities. Furthermore, a more comprehensive understanding of the physiological effects of MBS could enhance the prediction of surgical outcomes, support evidence-based decision-making for patient selection, and broaden the indications for surgical intervention [15,16].

Metabolomics, a key domain within the omics sciences and systems biology, entails the quantitative analysis of metabolites in biological samples. This methodological approach has proven promising in medical research for the identification of metabolic biomarkers and the elucidation of pathophysiological mechanisms across a range of diseases [16,17,18,19]. Within the scope of MBS, metabolomic studies primarily aim to accomplish two key objectives: first, to elucidate the physiological alterations driven by the metabolic impacts of the surgical procedure, and second, to discover preoperative biomarkers that can forecast postoperative metabolic results. These findings hold the potential to facilitate tailored treatment approaches and improve perioperative patient care by enabling predictive modeling and individualized management plans [20,21].

This narrative review presents the existing literature on metabolomics in the setting of MBS, with a particular focus on studies analyzing plasma or serum samples. The primary objective is to highlight the relevance of these investigations in elucidating postoperative metabolic adaptations and in identifying predictive biomarkers for key outcomes, such as T2D remission and weight loss.

## 2. Literature Search

For the preparation of this review, a comprehensive search was conducted in the PubMed database using the keywords “Bariatric Surgery” and “Metabolites”. The search was limited to publications from the past 25 years, resulting in a total of 649 articles published between 2000 and April 2025. We prioritized research articles, review papers, randomized controlled trials (RCTs), and meta-analyses. To ensure a thorough review, the references of these articles were also examined to identify additional relevant publications. Due to the volume of retrieved literature, it is recognized that not all relevant studies could be included or discussed in detail within the scope of this review.

## 3. Metabolomics as a Tool for Advancing Research in Metabolic Bariatric Surgery

### 3.1. Understanding the Dynamic Metabolic Landscape Through Metabolomics

Metabolomics offers an in-depth view of the dynamic and complex metabolic landscape within biological systems [22,23]. Metabolites are small organic molecules, typically weighing between 50 and 1500 Daltons, that participate in various biochemical processes as substrates, intermediates, or end products [22,24,25,26]. Common examples include sugars, lipids, fatty acids, amino acids (AAs), and nucleotides [22,24,25,26]. Closely related to metabolomics is the broader concept of metabonomics, which focuses on analyzing and comparing the overall metabolic profile of a sample or organism in response to various factors such as medications, diet, or disease. The term metabolome was first introduced in 1998 [27], with metabonomics and metabolomics being defined in 1999 and 2000, respectively [28,29]. By profiling a wide spectrum of small-molecule metabolites, metabolomics captures real-time biochemical changes in response to physiological processes, environmental stimuli, and disease states. This systems-level approach enables researchers to uncover complex biochemical interactions and regulatory networks that underlie health and disease, offering insights into key aspects of organismal function and potential therapeutic targets. Ultimately, metabolomics stands as a powerful tool for monitoring metabolic adaptations over time, supporting personalized medicine, and deepening our understanding of biological complexity [30,31,32,33].

### 3.2. Key Analytical Techniques for Metabolomic Studies in Metabolic Bariatric Surgery

Mass spectrometry (MS) is central in metabolomic research, offering high sensitivity in detecting metabolites by measuring their mass-to-charge ratio [34,35,36]. In MBS studies, MS is widely used to analyze plasma and urine samples, enabling the quantification of metabolic changes from baseline to postoperative states [37]. This technique facilitates the identification of diverse metabolite classes, such as lipids, AAs, bile acids (BAs), steroids, and endocannabinoids (ECs) [34,38,39]. Prior to MS analysis, chromatographic separation techniques, including liquid chromatography (LC) and gas chromatography (GC), are employed to resolve complex metabolite mixtures [40]. GC-MS is particularly suited for analyzing volatile compounds, whereas LC-MS is preferred for non-volatile metabolites in the context of MBS-related profiling [41].

Tandem mass spectrometry (MS/MS), typically coupled with LC as LC-MS/MS, improves the specificity of metabolite identification by inducing fragmentation of precursor ions and analyzing the resulting product ions. This allows for structural elucidation and more confident metabolite annotation, particularly in targeted metabolomics and biomarker validation studies [42]. Moreover, ultra-high-performance liquid chromatography (UHPLC) offers enhanced resolution and speed over conventional high-performance liquid chromatography (HPLC), making it especially advantageous for analyzing complex biological matrices [43]. Interestingly, UHPLC/MS has been applied to track temporal metabolic changes following MBS, contributing to the characterization of metabolic responses and surgical outcomes [44].

Alternative separation methods include capillary electrophoresis (CE) and ion mobility (IM) spectrometry. CE separates charged metabolites based on their size-to-charge ratio under an electric field and is well suited for detecting small polar compounds. CE-MS has been utilized to explore metabolomic differences across various MBS procedures [45]. On the other hand, IM spectrometry separates ions in the gas phase based on their drift through a buffer gas, offering high-resolution separation of isomeric and isobaric compounds [46]. When coupled with chromatographic methods, IM enhances compound coverage and resolution. This has been demonstrated in murine models assessing gut metabolomic differences across two biliary diversion procedures [47]. Lastly, nuclear magnetic resonance (NMR) spectroscopy presents a non-destructive, reproducible method for metabolite identification, relying on the magnetic properties of atomic nuclei. NMR enables metabolic profiling of both biofluids and tissue samples (using magic angle spinning for semi-solid specimens) and has been employed to detect changes in lipid and carbohydrate metabolism in plasma samples from MBS patients [48,49]. Table 1 presents an overview of the main methodologies used for metabolite analysis, highlighting their pros and cons.

## 4. Considerations Regarding Planning and Performing a Metabolomics Study

### 4.1. Study Design and Analytical Approaches in Metabolomics

Metabolomics studies can follow either a targeted or untargeted approach, or a combination of both. Targeted metabolomics involves the measurement of a predefined set of metabolites that are typically known or hypothesized to be associated with the condition under investigation or experimental manipulation. This approach is characterized by the precise identification of selected compounds and the use of analytical standards to achieve absolute quantification. In contrast, untargeted metabolomics is exploratory in nature and aims to detect as many measurable compounds as possible, without prior assumptions. It offers semi-quantitative data and is often used to discover novel biomarkers or metabolic pathways associated with the studied condition. Consequently, the selection between targeted and untargeted methods depends largely on the specific goals of the study. Both approaches have been employed in research on metabolite changes related to MBS [50].

Each technique has specific strengths and limitations [39,51,52]. MS methods offer high sensitivity, allowing the detection of metabolites in the nanomolar (nM) or even picomolar (pM) range, whereas NMR typically detects concentrations in the micromolar (μM) range. Moreover, combining MS with separation techniques improves selectivity, while NMR spectra are often affected by overlapping peaks, limiting their resolving power [53,54]. MS-based methods can measure a significantly greater number of metabolites, ranging from ~1000 to several thousand, compared to NMR, which typically detects up to ~200 metabolites. However, MS analysis requires labor-intensive sample preprocessing, destroys the sample, and is subject to ionization efficiency variability, which complicates quantification. In contrast, NMR allows metabolite quantification with minimal preprocessing, is non-destructive, and provides signal intensities directly proportional to metabolite concentrations. These features allow repeated analyses from the same sample and facilitate high reproducibility. As a result, NMR workflows are well-suited for automation, making them ideal for large-scale studies and for measuring metabolic fluxes in vitro and in vivo (fluxomics) [48,49].

### 4.2. Sample Handling, Extraction Protocols, and Quality Control

MS is susceptible to variability not related to the biological phenotype, particularly when samples are analyzed in separate batches or at different sites. This analytical variability, referred to as the batch effect, stems from technical differences in measurement procedures and poses a significant challenge for long-term or multicenter trials, which are common in MBS research [55]. Due to these complexities, including destructive analysis and challenging sample handling, MS is less suitable for fluxomics studies [53,54].

Sample size is another crucial factor in study planning. Existing public datasets or pilot studies can aid in estimating the required sample size using tools such as MetaboAnalyst (https://www.metaboanalyst.ca/) accessed on 25 April 2025) or metaX (http://metax.genomics.cn/) (accessed on 25 April 2025). Ideally, all MS samples should be analyzed in a single run [56]. However, in large-scale studies, this is often impractical. Therefore, special measures are required to detect and minimize inter-batch variability. Standardizing sample collection and storage is a vital first step. Some metabolites, such as dinucleotide redox cofactors and adenine nucleotides, are highly unstable and require specific conditions to preserve their integrity. If immediate analysis is not possible, minimizing time at room temperature before freezing is critical, as biochemical reactions continue post-collection. Storage at −80 °C or in liquid nitrogen is preferred to preserve the metabolic profile at the time of collection [56]. Furthermore, samples should be aliquoted to avoid freeze–thaw cycles that can alter metabolite composition, while also considering the volume needed for various analyses. Urine samples are more resilient to temperature fluctuations and freeze–thaw cycles and can be stored at −20 °C or −25 °C for several months without affecting metabolite profiles. Pre-analytical factor effects are comprehensively discussed by Stevens et al. [57].

The first step in MS analysis is sample extraction. Proteins must be removed from tissue, plasma, and serum samples to prevent interference during analysis. Two-phase liquid–liquid extraction methods are commonly used in metabolomics and lipidomics to separate polar and non-polar compounds. Solvent systems include methyl tert-butyl ether (MTBE)/methanol/water, chloroform/methanol/water, and dichloroform/methanol/water, with dichloromethane demonstrating similar lipid extraction efficiency to chloroform, while being less toxic. For large-scale studies, single-phase extractions using isopropanol or butanol/methanol mixtures have proven effective for extracting both complex lipids and polar metabolites from plasma [58].

A key advantage of MTBE or butanol/methanol over chloroform or dichloromethane is their lower density relative to water, allowing for easy pipetting of the top organic layer and minimizing contamination from salts, proteins, or sugars. However, extraction efficiency may vary depending on sample complexity. Three-phase extraction using hexane, methyl acetate, acetonitrile, and water enables the separation of metabolites into an aqueous phase (polar metabolites), a middle phase (glycerophospholipids), and a top phase (neutral lipids, such as triacylglycerols and cholesteryl esters). This method enhances the detection of low-abundance lipids with LC/MS and improves the separation of neutral and polar fatty acids using GC/MS [59]. Rakusanova et al. provided a detailed review of critical considerations in sample extraction, highlighting the importance of method selection, sample integrity, and contamination prevention [39].

In MS workflows, isotope-labeled internal standards play a vital role in quality control for large-scale analyses. These are added before sample preparation to monitor technical variability, retention times, mass accuracy, and ion suppression. Commercially available internal standard kits should be selected based on the metabolite classes of interest. Additionally, a pooled quality control sample—comprising material from all study samples—can be used for normalization. Standard reference materials, such as NIST SRM 1950 (a pooled plasma from 100 individuals representing the average U.S. population in 2006), support data harmonization across laboratories. During MS runs, pooled samples and reference materials are typically injected every 10–20 research samples for quality control. Randomizing the sample acquisition order is also essential to mitigate analytical drift and batch-related artifacts [52,60].

### 4.3. Data Acquisition, Processing, and Statistical Analysis

Targeted MS experiments are often conducted using low-resolution tandem mass spectrometry in multiple reaction monitoring (MRM) mode. In this configuration, a specific ion is selected in the first MS stage and fragmented, and the resulting product ions are filtered and detected in the second stage. This enhances selectivity, sensitivity, and quantification by reducing background noise and interference from co-eluting compounds. On the other hand, untargeted metabolomics commonly utilizes high-resolution tandem MS in either data-dependent acquisition (DDA) or data-independent acquisition (DIA) modes. In DDA, a full MS scan is first performed, and ions above a certain intensity threshold are isolated and fragmented within a narrow mass/charge (m/z) window. This improves the assignment of product ions to precursor ions but limits the detection of low-abundance metabolites [61]. DIA, by contrast, uses broader m/z windows to include all precursor ions within a range, enhancing metabolite coverage. However, DIA spectra are more complex and harder to interpret due to overlapping signals [39].

Following MS acquisition, raw data undergo preprocessing steps such as mass detection, chromatogram construction, deisotoping, peak alignment, and gap-filling. While vendor-provided software is often used, open-source tools and packages written in languages such as R or Matlab are also available [62]. Notably, studies have shown significant variation in results depending on the software used to process identical raw LC/MS datasets [63]. Therefore, careful quality assurance, including the use of reference standards, is necessary to validate preprocessing outcomes [39].

Several comprehensive databases, such as METLIN Gen2 (metlin.scripps.edu), contain molecular and MS/MS spectral data that assist in metabolite identification [39]. Additionally, computational tools exist to predict unknown compound structures from MS spectra [64]. Before statistical analysis, normalization is required to correct for technical and biological variability, including sample handling, signal drift, and batch effects. Quality control is essential to support effective data normalization [52].

Metabolomics studies related to MBS often integrate pathway databases to investigate the effects of surgical intervention on metabolism [65]. Advanced statistical and bioinformatic tools are critical for interpreting complex metabolomic datasets [66]. Commonly used multivariate techniques include principal component analysis (PCA) and partial least squares discriminant analysis (PLS-DA) [67]. PCA is an unsupervised dimensionality reduction method that can reveal clusters of related metabolites, often corresponding to specific clinical phenotypes or biochemical pathways. PLS-DA is a supervised classification technique that identifies metabolites associated with a binary outcome variable, such as surgical response. Unlike traditional multivariate regression, PLS-DA accounts for multicollinearity and maximizes covariance with the outcome. Orthogonal PLS-DA (OPLS-DA) further separates variation related to metabolites from variation unrelated to the primary response variable. It is important to note that both PLS-DA and OPLS-DA are susceptible to overfitting. Therefore, when using metabolomics data to predict MBS outcomes, datasets should be divided into training and testing sets. If sample size limits this option, cross-validation techniques should be employed to ensure the resulting model is robust and unbiased [62,63].

## 5. Metabolomic Changes Following Metabolic Bariatric Surgery

### 5.1. Amino Acid Derivatives

AAs serve as essential building blocks of peptides and proteins, making them key molecules in various biological functions. In the field of metabolomics, AAs have garnered significant attention due to their pivotal involvement in metabolic pathways that regulate energy balance, immune responses, and cellular function. Their levels and profiles are often analyzed to understand metabolic health, particularly because alterations in AA concentrations have been linked to conditions such as obesity and insulin resistance (IR). Exploring AA metabolism enhances our understanding into the underlying mechanisms of metabolic disorders and can aid in identifying potential biomarkers for disease states and therapeutic targets [10,68,69].

#### 5.1.1. Role of Branched-Chain Amino Acids (BCAAs) in Obesity and Metabolic Disease

Research by Wang et al. demonstrated that elevated circulating levels of leucine, isoleucine, and valine are strong predictive biomarkers for the future development of T2D [70]. These AAs are collectively referred to as BCAAs due to their shared structural characteristic of having a branched side chain [71]. Additionally, they follow similar initial pathways during their catabolism, reflecting their close biochemical relationship and their potential role as active mediators of IR [70]. A separate group of researchers emphasized that impaired BCAA catabolism may contribute to altered mitochondrial substrate flux and redox imbalance in insulin-resistant states [68]. However, Mihalik et al. showed that while young individuals with obesity and T2D exhibit increased lipid oxidation and altered AA profiles, their metabolomic signatures do not suggest impaired mitochondrial oxidative metabolism, but rather point to an early adaptive metabolic response [72].

Some studies suggest that elevated levels of BCAAs under metabolic stressors such as T2D and starvation may be partly due to impaired catabolism, possibly involving mitochondrial dysfunction. BCAAs, particularly leucine, can activate the mammalian target of rapamycin (mTOR) signaling, which in turn may disrupt insulin signaling and impair glucose homeostasis [73,74,75,76]. While higher dietary intake of BCAAs has been associated with increased risk of IR and T2D, findings remain inconsistent. It has been proposed that both exogenous factors (e.g., excessive dietary BCAA intake) and endogenous metabolic impairments (e.g., mitochondrial dysfunction or reduced BCKD activity) may act synergistically, contributing to metabolic dysregulation. This interplay may help explain how chronic elevations in circulating BCAAs could worsen insulin signaling, promote adiposity, and increase the risk of glucose intolerance over time [77].

MBS has been shown to reduce circulating BCAA levels. Tan et al. reported that BCAA oxidation is elevated in adults with morbid obesity but decreases after sleeve gastrectomy (SG), in parallel with improved insulin sensitivity and reduced plasma BCAA concentrations [78]. Animal studies suggested that the postoperative decline in plasma BCAAs may be mediated by increased secretion of fibroblast growth factor 21 (FGF21), a hormone that promotes mitochondrial BCAA oxidation [79,80]. This enhanced catabolic activity alleviates metabolic stress, improves mitochondrial efficiency, and contributes to restored metabolic balance. Long-term follow-up studies across diverse populations [81,82,83] have demonstrated that the reduction in circulating BCAAs persists well beyond the early postoperative period. These sustained changes are associated with durable improvements in insulin sensitivity, lipid profiles, and systemic inflammation. The prolonged effect appears to be largely mediated by hormonal mechanisms, particularly the action of FGF21, which facilitates mitochondrial BCAA utilization and promotes metabolic homeostasis [80].

#### 5.1.2. Aromatic Amino Acids (AAAs) and Their Link with Obesity and Glucose Dysregulation

Elevated levels of the AAAs phenylalanine and tyrosine have been associated with the development of IR and T2D, serving as early indicators of metabolic dysfunction across diverse ethnic populations [70,84,85,86,87]. Serum phenylalanine levels not only serve as diagnostic markers but may also reflect hepatic metabolic capacity. The observed reduction in phenylalanine after MBS coincides with improvements in liver function, which could indicate enhanced liver detoxification. These changes are often accompanied by reduced systemic inflammation and improved glucose homeostasis, both closely linked to decreased IR in obesity [88].

Obesity activates the kynurenine pathway, the main route of tryptophan catabolism, resulting in elevated levels of pro-inflammatory and neurotoxic metabolites, such as quinolinic and xanthurenic acids [89,90,91]. These compounds promote systemic inflammation, oxidative stress, and IR, perpetuating metabolic dysfunction. After MBS, this pathway is downregulated, resulting in decreased levels of these deleterious metabolites [89,90,91,92]. Another key pathway involves dopamine and its precursors within the gastrointestinal tract. Eisenhofer et al. identified the gut as a major site of dopamine synthesis, with 3,4-Dihydroxy-L-phenylalanine (L-DOPA) acting as a central intermediate in local catecholamine metabolism [93]. Gut-derived dopamine may function as an anti-incretin, influencing glucose homeostasis, particularly after gastrointestinal procedures such as bypass surgery [94]. In rodent models, Korner et al. showed that foregut tyrosine metabolism (including L-DOPA biosynthesis) directly affects glucose tolerance [95]. Supporting this, metabolomic studies have identified L-DOPA-related metabolites as predictors of T2D remission post-surgery [96]. In parallel, obesity is associated with impaired central dopaminergic signaling, characterized by downregulated dopamine receptor availability due to chronic overstimulation of the reward system. Notably, Roux-en-Y gastric bypass (RYGB) appears to partially reverse this dysfunction, normalizing receptor levels and potentially contributing to postoperative improvements in reward processing and metabolic regulation [97,98,99].

#### 5.1.3. Non-Classical Amino Acids and Their Metabolic Significance

Obesity-associated declines in glycine and serine compromise antioxidant capacity, whereas MBS may restore their concentrations, thereby improving redox balance and insulin sensitivity [100,101,102,103]. Research also supports that plasma citrulline levels increase significantly twelve months post-surgery, with greater elevations observed after gastric bypass than SG, despite comparable benefits in body weight and fat mass [104]. Polyamines synthesized from AAs, like ornithine and arginine, are integral to cellular proliferation, transcriptional regulation, and inflammatory processes [105]. Elevated levels of the polyamines spermidine and spermine have been implicated in the pathophysiology of obesity-associated chronic inflammation and IR [106,107]. MBS may promote reductions in inflammatory markers and improvements in metabolic status by lowering systemic polyamine concentrations [108]. Table 2 depicts the main alterations in circulating AA levels following MBS, along with their metabolic implications and potential clinical outcomes.

### 5.2. Lipid Derivatives

MBS induces key metabolic changes, including alterations in various lipid-derived molecules that play critical roles in energy homeostasis, inflammation, and cell signaling. Studies have shown that postoperative shifts in lipid derivatives, such as acylcarnitines, ceramides, BAs, and ketone bodies, are closely associated with improvements in metabolic health and insulin sensitivity [109,110].

#### 5.2.1. Fatty Acids, Membrane Lipids, and Energy-Related Lipid Metabolites

The interplay between lipid metabolism and obesity-related metabolic disorders has gained increasing attention [111]. Central to this is carnitine-dependent mitochondrial fatty acid oxidation; when impaired, it leads to the accumulation of acylcarnitine intermediates, which are elevated in individuals with obesity and T2D [112,113]. Laferrière et al. demonstrated that gastric bypass induces specific changes in lipid metabolism independent of weight loss, notably enhancing acylcarnitine profiles and glycemic control; these effects are not replicated by caloric restriction alone [114]. Supporting this, postprandial metabolomics showed increased acylcarnitine levels after meals, suggesting improved mitochondrial flexibility in nutrient handling [115]. These shifts reflect enhanced fatty acid oxidation and reduced metabolic stress, a finding further supported by broader profiling studies showing that MBS remodels serum fatty acids and acylcarnitines, enhancing mitochondrial function [116,117,118,119]. Acylcarnitine fluctuations post-RYGB also indicate transient but dynamic improvements in substrate utilization and respiratory activity during the early postoperative phase [120].

Phospholipids, key structural elements of cell membranes, have recently been implicated in IR pathophysiology [121]. Levels of phosphatidylcholines and phosphatidylethanolamines, the most abundant mammalian phospholipids, have been shown to decrease significantly after RYGB and SG, though the clinical implications of these changes remain unclear [122,123]. On the other hand, ceramides, a class of bioactive sphingolipids, play a central role in lipid-induced metabolic dysfunction by impairing insulin signaling through protein kinase B (Akt) inhibition [124]. Their plasma levels are elevated in obesity and T2D, correlating with IR severity [125]. Experimental inhibition of ceramide synthesis improves insulin sensitivity and alleviates lipotoxic stress in models of obesity and high saturated fat intake [126]. In humans, post-MBS reductions in ceramide subspecies have been linked to improved insulin sensitivity, lipid homeostasis, and cardiovascular risk markers, including the apolipoproteinB100/apolipoproteinA1 (ApoB100/ApoA1) ratio [127,128].

Ketone bodies (β-hydroxybutyrate, acetoacetate, and acetone), also products of mitochondrial fatty acid oxidation, are typically elevated in fasting or carbohydrate-restricted states. Obesity disrupts their metabolism, reflecting mitochondrial inefficiency and impaired metabolic flexibility [129]. After MBS, their increase signals a metabolic shift toward alternative fuel utilization and enhanced oxidative capacity. However, while concentrations commonly increase in the early postoperative phase, they tend to decline over time as energy balance is reestablished and metabolic homeostasis is progressively restored [130]. Concurrently, changes in the tricarboxylic acid cycle (TCA), such as elevated citrate and reduced pyruvate, suggest metabolic reprogramming to optimize energy production. Increases in citrate, succinate, and malate after SG and RYGB further support improved mitochondrial function [131].

#### 5.2.2. Bile Acids

BAs are synthesized from cholesterol in the liver, stored in the gallbladder, and released into the duodenum in response to intestinal stimuli. Approximately 95% are reabsorbed in the terminal ileum and returned to the liver via the portal vein, constituting the enterohepatic circulation. The remaining fraction enters systemic circulation, contributing to serum BA levels. Elevated serum BAs may arise from increased hepatic synthesis, enhanced ileal reabsorption, or reduced fecal excretion [132]. Following RYGB, increased BA signaling has been linked to enhanced lipid and mitochondrial metabolism, as reflected by postoperative reductions in serum acylcarnitine concentrations [133]. Proteomic and metabolomic studies further support decreases in acylcarnitines and other lipid metabolites after surgery, in association with reduced adiposity and improved insulin sensitivity [134]. In addition to facilitating lipid absorption, BAs function as signaling molecules regulating glucose and energy metabolism, primarily through activation of the farnesoid X receptor (FXR) and G-protein-coupled bile acid receptor 1 (GPBAR1/TGR5) [135,136,137].

Research supports that MBS increases circulating levels of both primary and secondary BAs [138,139,140], which may in turn contribute to postoperative metabolic improvements through the activation of FXR and TGR5 pathways [141,142]. In a study by Wahlström et al., BA kinetics were assessed in individuals with obesity, with or without T2D, before and after MBS. Postoperative changes included elevated fasting plasma BA levels, decreased fecal BA excretion, increased postprandial fibroblast growth factor 19 (FGF19), and reduced levels of 7α-hydroxy-4-cholesten-3-one (C4)—a surrogate marker of hepatic BA synthesis. The study also reported elevated fasting levels of 6α-hydroxylated BAs, particularly hyodeoxycholic acid, which correlated with T2D remission in an independent cohort [143]. However, the long-term implications of altered secondary BA synthesis, potentially reflecting gut microbiota shifts, remain to be clarified [144].

#### 5.2.3. Endocannabinoids

The EC system plays a key role in regulating energy balance, metabolism, and obesity-related disorders. Early studies demonstrated its involvement in glucose and lipid metabolism, implicating ECs in the pathogenesis of T2D and metabolic syndrome [145]. Plant-derived cannabinoids have been involved in the development and progression of T2D and its complications [146]. Moreover, endogenous cannabinoids may influence energy homeostasis by activating central orexigenic pathways that increase appetite and promoting peripheral lipogenesis, which enhances fat storage [147]. In individuals with abdominal obesity, the EC system’s activity becomes dysregulated in both adipose and peripheral tissues, with elevated EC levels being associated with increased fat accumulation and IR [148]. As detailed by Silvestri and Di Marzo, this dysregulation contributes to energy imbalance and disease progression, positioning ECs as an attractive therapeutic target [149]. Subsequent studies have identified circulating EC levels as potential biomarkers of the EC system’s overactivation in severe obesity, linking them to key metabolic disturbances [150]. Interestingly, evidence suggests that selective serotonin reuptake inhibitors (SSRIs) may indirectly modulate the EC system’s activity and energy balance through serotonin 2C receptor-mediated effects on gastrointestinal motility [151].

Recent research indicates that MBS modulates the EC system’s activity. Circulating levels of key ECs, including arachidonic acid, 2-arachidonoylglycerol, and anandamide, decrease following procedures such as gastric bypass, and these reductions are associated with improved metabolic homeostasis, insulin sensitivity, and reduced inflammation [152,153]. Notably, decreased EC levels post-MBS have also been linked to enhanced coronary circulatory function in individuals with obesity [153]. Importantly, the EC system’s dysregulation may precede the clinical onset of obesity. In a whole-body PET study of healthy young adults, those at high risk for obesity exhibited reduced cannabinoid type 1 receptor (CB1R) availability in adipose tissue compared to low-risk individuals. CB1R availability in both abdominal fat and the brain was positively associated with insulin sensitivity, highlighting early alterations in the EC system’s function that may contribute to metabolic disease development [154]. Table 3 represents an overview of the metabolic and lipid pathway modifications induced by MBS, highlighting the underlying mechanisms and their systemic impacts on energy homeostasis, inflammation, insulin sensitivity, and cardiovascular health.

### 5.3. The Interplay Between Intestinal Microbiota and Their Metabolic Outputs

The gut microbiota, composed of a vast community of commensal microorganisms, plays a pivotal role in host metabolic health [155,156]. Alterations in its composition have been linked to obesity, chronic inflammation, and metabolic disorders such as T2D and dyslipidemia [157,158,159]. The gut microbiota, metabolites, and host immunity are intricately interconnected, with microbial-derived compounds playing essential roles in regulating metabolic and immune functions [160,161,162]. Post-MBS, significant alterations in the composition of the gut microbiota lead to changes in various metabolites, including trimethylamine N-oxide (TMAO), phenyl sulfate, and p-cresol sulfate, which can influence cardiovascular and metabolic health. For example, levels of phenyl sulfate and p-cresol sulfate, both products of microbial metabolism of AAAs, have been associated with increased cardiovascular risk and metabolic inflammation. Additionally, TMAO levels, which tend to increase within a year after surgery, are linked to atherogenesis. These microbial metabolites also contribute to the improvement of insulin sensitivity and metabolic regulation, indicating that modifications in the gut microbiota and its metabolic outputs may be crucial in the metabolic benefits observed following bariatric procedures [160,161,162].

In a prospective observational cohort study, researchers enrolled adults with obesity scheduled to undergo SG and conducted follow-up assessments six months postoperation. The study aimed to evaluate changes in gut microbial composition and related metabolites by comparing the bariatric cohort with a healthy control group. In comparison to the healthy control group, individuals in the MBS group exhibited significantly elevated baseline plasma levels of the short-chain fatty acid (SCFA) acetate [163]. In the gastrointestinal tract, the gut microbiota metabolizes tryptophan into various indole compounds, including indole-3-acetic acid (IAA), indole, and indole-3-propionic acid (IPA) [164]. Dysbiosis associated with obesity leads to decreased production of these microbial metabolites, which are known to be inversely linked to systemic inflammation [165]. Importantly, reduced circulating levels of IPA have been connected to a higher risk of developing T2D [166]. After MBS, there is an increase in the levels of 3-indoxyl sulfate, IAA, and IPA, with elevated IPA levels being associated with improved insulin sensitivity [167].

**Figure 1:**
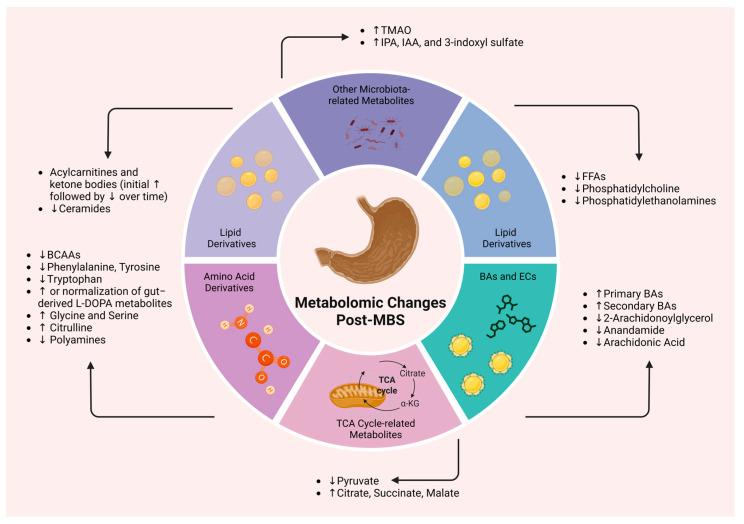
Key metabolomic alterations following metabolic bariatric surgery. Abbreviations: a-KG: alpha-ketoglutarate; BAs: bile acids; BCAAs: branched-chain amino acids; ECs: endocannabinoids; FFAs: free fatty acids; IAA: indole-3-acetic acid; IPA: indolepropionic acid; MBS: metabolic bariatric surgery; TCA: tricarboxylic acid (cycle); TGs: triglycerides; TMAO: trimethylamine N-oxide; ↑: increase; ↓: decrease. Created in BioRender. Kounatidis, D. (2025) https://BioRender.com/o93j3mc. Assessed on 24 June 2025.

Bariatric procedures may induce significant alterations in the gut microbial ecosystem, affecting both the temporal dynamics and spatial organization of intestinal communities post-surgery [168]. These microbiota shifts are increasingly recognized as contributors to the metabolic benefits of the intervention and may hold prognostic value for postoperative outcomes. Baseline microbiota profiles prior to surgical intervention may serve as predictive markers of individual responsiveness, while early postoperative modifications characterized by an enrichment of beneficial microbial taxa may correlate with more favorable weight-loss trajectories and metabolic outcomes [169]. The sustained weight reduction and alleviation of obesity-related comorbidities achieved through MBS may be, in part, mediated by these microbiome changes [170,171]. Research exploring the microbiome’s role post-MBS extends beyond compositional analyses, delving into functional aspects of microbial communities and their metabolic activities [172]. This focus has prompted investigation into the microbiota’s potential as a biomarker for surgical success, particularly in relation to T2D remission and long-term weight maintenance [169,172]. Figure 1 illustrates the principal metabolomic changes observed after MBS, based on current evidence.

## 6. Procedure-Specific Metabolomic Signatures in Metabolic Bariatric Surgery

Vaz et al. conducted a systematic review and meta-analysis that specifically examined parallel-arm studies comparing the metabolomic profiles elicited by various MBS procedures. A synthesis of 47 eligible studies revealed that the most affected metabolite classes following MBS were AAs, lipids, energy metabolism-related compounds, and gut-microbiota-associated metabolites. Notably, elevated pre-surgical levels of certain lipids, such as phospholipids, long-chain fatty acids, and BAs, were linked to improved T2D remission outcomes [173]. Moreover, in an observational study, Gralka et al. identified a distinct metabolomic profile in individuals with severe obesity, characterized by elevated levels of BCAAs, AAAs, energy-related metabolites, and gut-microbiota-derived compounds. MBS, regardless of type (SG, proximal RYGB, and distal RYGB), significantly altered this profile in a dynamic and procedure-specific way, bringing it closer to that of normal-weight individuals and indicating substantial shifts in host–microbiome interactions [174]. In a separate study, Jarak et al. found no evidence of impaired short-term nutrient absorption following RYGB with a long (2 m) biliopancreatic limb compared to the standard procedure. Although postprandial metabolite profiles were largely similar between groups, acetate levels were significantly elevated in the long-limb group at 120 min, which may play a role in the observed metabolic benefits [175].

Restrictive and malabsorptive MBS procedures exert distinct effects on the serum lipidome in individuals with obesity. Restrictive surgeries, such as SG, primarily lower food intake and are associated with reductions in triglycerides (TGs) and LDL cholesterol (LDL). In contrast, malabsorptive procedures like RYGB induce broader alterations in lipid metabolism, impacting various lipid species including phospholipids, sphingolipids, and sphingomyelins [176,177,178]. Fasting and postprandial metabolomic responses were also explored in a cross-sectional study comparing two malabsorptive procedures: single anastomosis duodenoileostomy with sleeve gastrectomy (SADI-S) and biliopancreatic diversion with duodenal switch (BPD-DS). The study found that postprandial levels of BCAAs were significantly higher following SADI-S compared to BPD-DS [179].

Predicting weight loss or regain following MBS is essential for effective patient management. Although the majority of patients achieve long-term weight loss, a significant proportion experience weight regain, ranging from 10 to 20% after RYGB and 5 to 70% after SG [180,181,182]. Preoperative serotonin levels and the serotonin-to-5-hydroxytryptophan ratio predict slow weight loss at six months post-SG [182]. Untargeted metabolomic analysis reveals that patients with sustained weight loss exhibit distinct profiles, characterized by reduced fatty acid metabolites and increased glycine levels compared to those experiencing weight regain after six months [183]. Interestingly, elevated total cysteine levels at two years post-RYGB have been associated with subsequent weight gain [184].

## 7. Metabolic Bariatric Surgery Versus Incretin-Based Pharmacological Weight-Loss Interventions

Glucagon-like peptide-1 receptor agonists (GLP-1 RAs) represent a relatively recent class of antidiabetic agents that have significantly enhanced glycemic control in patients with T2D, while also providing cardiovascular benefits and promoting weight loss [185]. More recently, tirzepatide, a dual glucose-dependent insulinotropic polypeptide (GIP)/GLP-1 RA, has been introduced into clinical practice, demonstrating notable efficacy, with even more potent favorable effects on weight reduction [186]. An increasing number of studies have compared these incretin-based therapies with MBS. Research suggests that both approaches may offer comparable efficacy in glycemic control; however, MBS may result in greater reductions in body weight and body mass index (BMI) [187]. Cardiovascular outcomes remain an area of debate, with limited and conflicting data. A recent meta-analysis supports the superiority of MBS over GLP-1 RAs in cardiovascular protection [188], whereas other comparative data suggest that tirzepatide may offer greater benefits, including a greater reduction in major adverse kidney events (MAKEs) (HR, 0.375; 95% CI, 0.336–0.419; *p* < 0.0001) [189]. Nevertheless, conclusions remain provisional, as current evidence derives from observational studies, and RCTs are still lacking.

The metabolomic alterations induced by MBS versus those from incretin-based interventions represent a developing area of research. MBS, particularly RYGB and SG, triggers rapid and substantial changes in the metabolic profile, often distinct from those associated with pharmacological treatments. These post-surgical changes reflect not only weight loss but also marked modifications in gut physiology, nutrient absorption, and hormonal regulation. These effects are accompanied by marked shifts in BA profiles, lipid metabolism, and AA composition, reflecting changes in gut anatomy, nutrient absorption, and entero-endocrine signaling—factors closely associated with improved insulin sensitivity and glucose homeostasis [190,191,192].

In contrast, GLP-1 RAs primarily influence glycemic regulation and appetite suppression, resulting in more targeted and modest metabolomic effects. Although there is some overlap, such as alterations in AA and lipid metabolism, MBS elicits unique signatures involving gastrointestinal hormones and nutrient processing. Some shared metabolomic responses may reflect general consequences of weight loss or caloric restriction, rather than being specific to the intervention [190,191,192]. Further comparative studies are warranted to elucidate these distinctions and evaluate the potential of metabolomic profiling to differentiate between weight-loss modalities and their respective biological mechanisms.

## 8. Limitations, Challenges, and Research Gaps in Contemporary Metabolomic Studies Within Metabolic Bariatric Surgery

Despite notable progress in metabolomics, particularly in experimental and clinical research contexts, its translation into routine clinical practice remains fraught with substantial challenges. A primary barrier lies in the lack of standardization across analytical workflows, which significantly hampers reproducibility and inter-study comparability. Variations in sample collection, preparation, instrument calibration, and data acquisition methods across laboratories introduce inconsistencies that undermine biomarker validation efforts and preclude the establishment of universally accepted metabolic signatures. The analytical complexity of metabolomics itself poses intrinsic challenges. Although thousands of features can be detected in biological samples, only a minority can be identified as known metabolites [50,69,110]. The vast majority remain unannotated, limiting biological interpretability and restricting their clinical utility. This issue is compounded by the inadequate standardization of data processing pipelines, which often lack robustness in feature detection, annotation, and integration with other omic datasets. As a result, metabolomic outputs, often consisting of high-dimensional molecular signatures with subtle abundance changes, remain difficult to distill into clinically actionable information. Moreover, the requirement for sophisticated, high-cost instrumentation further restricts accessibility, posing a major barrier to clinical implementation [50,69,110].

In the context of MBS, methodological limitations are further enhanced by the influence of comorbid conditions such as T2D and dyslipidemia, which modulate metabolic profiles both pre- and postoperatively. Additionally, inter-individual variability, shaped by genetic background, environmental exposures, lifestyle, and gut microbiome composition, necessitates a shift toward precision medicine strategies [69,193]. Integrating genomics, proteomics, microbiome, and metabolomic data through multi-omics platforms may enhance the resolution of metabolic adaptations following MBS and support individualized therapeutic approaches. Another limitation is the heterogeneity of study populations and the absence of appropriate stratification. Unstratified cohorts obscure the physiological effects attributable to MBS itself, diluting the specificity of metabolomic findings. Stratified or individualized analyses are therefore essential to delineate distinct metabolic responses and identify subpopulations that may derive differential benefit from surgical interventions. Additionally, dietary intake and concurrent pharmacological treatments are major confounders of metabolomic signatures and must be closely controlled to enhance data reliability and reproducibility [194].

Temporal aspects of metabolic remodeling post-MBS remain under-characterized due to the predominance of cross-sectional study designs, small sample sizes, and short follow-up durations. Such limitations constrain the ability to capture dynamic metabolic trajectories and obscure long-term effects [195,196,197,198]. Longitudinal, multicenter studies with ethnically and clinically diverse populations are needed to track metabolic adaptations over extended periods, enabling the identification of early predictive biomarkers and assessment of sustained therapeutic impact. From an analytical standpoint, the metabolome’s intrinsic dynamism and the bidirectional interplay between metabolites complicate data interpretation. Advanced multidimensional methodologies, including machine learning and systems biology approaches, are increasingly necessary to disentangle complex metabolic networks and uncover latent patterns. While several predictive models integrating metabolomic and clinical data are under development, their clinical validation is still limited. Enhancing interoperability, cost-efficiency, and integration into electronic health record systems will be critical for future clinical deployment [195,196,197,198].

There are also notable research gaps in the specific metabolite classes investigated in MBS studies [199,200,201]. Modified amino acids (e.g., gamma-glutamyl and methylated derivatives), kynurenine pathway metabolites, oxidized lipid species, bile acid subtypes, and gut-microbiota-derived SCFAs remain insufficiently characterized, despite their putative roles in metabolic reprogramming, immune modulation, and inflammation resolution [202,203]. Furthermore, emerging areas such as exosomal nucleic acids and trace-element-associated metabolomics (e.g., zinc, magnesium, iron) offer promising perspectives for biomarker discovery but have received limited attention [204,205,206].

Finally, the absence of functional correlation between metabolomic alterations and clinical endpoints remains a fundamental gap. Incorporating physiological assessments, such as hormonal profiling, insulin sensitivity tests, and imaging modalities, could bridge this divide and provide mechanistic insights into the metabolic benefits of surgery. Ultimately, translating metabolomic discoveries into clinically meaningful tools will require not only methodological refinement but also a concerted effort to align biomolecular data with functional and phenotypic outcomes [199,200,201].

## 9. Conclusions

This review provides a comprehensive description of the impact of MBS on the metabolomic profiles of patients with obesity, elucidating how various surgical interventions generate distinct metabolomic signatures. These variations in metabolomic profiles may help explain the heterogeneity observed in surgical outcomes. Preoperative metabolomic fingerprints have been identified as potentially prognostic biomarkers for predicting responses to weight loss and remission of T2D. Specifically, elevated preoperative levels of lipids, including phospholipids, long-chain fatty acids, and BAs, have been associated with postoperative remission of T2D. These findings suggest that the metabolic state of patients prior to surgery may significantly influence their postoperative weight loss and overall metabolic health outcomes. Although initial investigations into identifying predictive biomarkers for surgical outcomes show promise, further research is crucial to uncover robust biomarkers and develop validated predictive models. Improved preoperative prediction of metabolic responses can enhance patient selection and optimize perioperative management. Additionally, further exploration of the short- and long-term systemic adaptations following MBS will clarify the underlying mechanisms of weight loss and may lead to the discovery of novel therapeutic targets for obesity.

## Figures and Tables

**Table 1:** An overview of the main methodologies used for metabolite analysis: advantages and limitations.

Technique	Advantages	Disadvantages
Nuclear Magnetic Resonance (NMR) Spectroscopy [48,49]	- High reproducibility - Simple and minimal sample preparation - Broad metabolite coverage (both polar and non-polar) - Straightforward metabolite identification	- Low sensitivity - Limited resolution
Gas Chromatography–Mass Spectrometry (GC-MS) [41,49]	- Excellent sensitivity - Superior resolution compared to NMR - Reliable metabolite identification using spectral libraries - Detects volatile compounds (both polar and non-polar)	- Requires extensive sample preparation - Lower reproducibility compared to NMR
Capillary Electrophoresis–Mass Spectrometry (CE-MS) [45,49]	- Higher resolution than NMR - Good sensitivity - Suitable for polar metabolites	- Medium level of sample preparation needed - Less reproducible than NMR - Difficult metabolite identification
High-Performance Liquid Chromatography–Mass Spectrometry (HPLC-MS) [44,49]	- High sensitivity - Improved resolution compared to NMR - Can separate both polar and non-polar metabolites depending on column type	- Requires moderate sample preparation - Lower reproducibility than NMR - Challenging metabolite identification due to incomplete databases

**Table 2:** Key postoperative changes in amino acid profiles following metabolic bariatric surgery and their clinical impact.

Amino Acids	Post-MBS Change	Impact and Outcomes
Branched-Chain Amino Acids [73,74,75,76,77,78,79,80]	↓	Reduction correlates with improved mitochondrial function, insulin sensitivity, and metabolic health; dietary intake linked to metabolic disorders
Phenylalanine and Tyrosine [70,84,85,86,87,88]	↓	Associated with improved hepatic function, reduced inflammation, and better glucose regulation; early biomarkers of metabolic decline
Tryptophan Pathway Metabolites [89,90,91,92]	↓	Leads to reduced systemic inflammation and improved insulin sensitivity, aiding metabolic recovery
Dopamine Precursors (L-DOPA) [93,94,95,96]	↑ or normalized	Restores receptor levels, potentially improving reward processing and glucose regulation, contributing to T2D remission
Glycine and Serine [104]	↑	Enhances antioxidant capacity, reduces oxidative stress, and alleviates IR
Citrulline [107]	↑	Indicates improved intestinal function, with positive effects on metabolic health

Abbreviations: IR: insulin resistance; L-DOPA: 3,4-Dihydroxy-L-phenylalanine; T2D: type 2 diabetes; ↑: increase; ↓: decrease.

**Table 3:** Metabolic and lipid pathway modulations post-metabolic bariatric surgery: Mechanisms and systemic impacts.

Category	Post-MBS Changes	Impact and Outcomes
Acylcarnitines and Fatty Acid Oxidation [112,113,114,115,116,117,118,119,120]	- ↑ acylcarnitine profiles - ↑ postprandial acylcarnitine response - ↑ substrate utilization - ↓ in acylcarnitine levels over time	- ↑ mitochondrial flexibility and function - ↑ fatty acid oxidation - ↓ metabolic stress - ↑ glycemic control and insulin sensitivity
Phospholipids [121,122,123]	- ↓ phosphatidylcholines and phosphatidylethanolamines (particularly after RYGB and SG)	- May reflect membrane composition and lipid remodeling - Possible role in insulin resistance regulation - Clinical significance still unclear
Ceramides [129,130,131]	- ↓ plasma ceramide subspecies	- ↑ insulin signaling and sensitivity - ↓ lipotoxicity - ↓ ApoB100/ApoA1 ratio
Ketone Bodies and TCA Cycle [44,129,130,131]	- ↑ β-hydroxybutyrate, acetoacetate, and acetone (although they tend to ↓ over time) - ↑ citrate, succinate, and malate - ↓ pyruvate	- ↑ metabolic flexibility - ↑ mitochondrial oxidative capacity - ↑ insulin sensitivity
Bile Acids [138,139,140,141,142,143,144]	- ↑ fasting and postprandial circulating BAs - ↓ fecal BA excretion - ↑ hyodeoxycholic acid - ↓ C4 levels	- Enhanced lipid and mitochondrial metabolism - Improved glucose metabolism - T2D remission in some individuals
Endocannabinoids [152,153,154]	- ↓ circulating ECs - Modulation of EC system activity	- ↓ inflammation and fat accumulation - Improved energy balance and metabolic homeostasis - Enhanced coronary circulatory function - Potential early detection and intervention target for metabolic dysfunction

Abbreviations: ApoA1: apolipoprotein A1; ApoB100: apolipoprotein B100; BA: bile acid; C4: 7α-hydroxy-4-cholesten-3-one; EC: endocannabinoid; RYGB: Roux-en-Y gastric bypass; SG: sleeve gastrectomy; T2D: type 2 diabetes; TCA: tricarboxylic acid cycle; ↑: increase; ↓: decrease.

## Data Availability

No new data were created or analyzed in this study. Data sharing is not applicable to this article.

## Abbreviations

AA: amino acid; AAA: aromatic amino acid; a-KG: alpha-ketoglutarate; Akt: protein kinase B; ApoA1: apolipoprotein A1; ApoB100; apolipoprotein B100; BA: bile acid; BCAAs: branched-chain amino acids; BMI: body mass index; BPD-DS: BPD with duodenal switch; BPL: biliopancreatic limb; CB1R: cannabinoid type 1 receptor; CE: capillary electrophoresis; CVD: cardiovascular disease; DDA: data-dependent acquisition; DIA: data-independent acquisition; EC: endocannabinoid; FGF: fibroblast growth factor; FXR: farnesoid X receptor; GC: gas chromatography; GIP: glucose-dependent insulinotropic polypeptide; GLP-1: glucagon-like peptide-1; GPBAR1: protein-coupled bile acid receptor 1; HOMA: homeostatic model assessment; HPLC: high-performance liquid chromatography; IAA: indole-3-acetic acid; IM: ion mobility; IPA: indole-3-propionic acid; IR: insulin resistance; LAGB: laparoscopic adjustable gastric banding; LC: liquid chromatography; LDL: low-density lipoprotein; L-DOPA: 3,4-Dihydroxy-L-phenylalanine; MAKE: major adverse kidney event; MASLD: metabolic-dysfunction-associated steatotic liver disease; MBS: metabolic bariatric surgery; MRM: multiple reaction monitoring; MS: mass spectrometry; MS/MS: tandem mass spectrometry; MTBE: methyl tert-butyl ether; mTOR: mammalian target of rapamycin; NMR: nuclear magnetic resonance; NO: nitric oxide; OPLS-DA: orthogonal partial least squares discriminant analysis; PCA: principal component analysis; PCOS: polycystic ovary syndrome; PLS-DA: partial least squares discriminant analysis; RCT: randomized controlled trial; RYGB: Roux-en-Y gastric bypass; SADI-S: single anastomosis duodenoileostomy with SG; SCFAs: short-chain fatty acids; SG: sleeve gastrectomy; T2D: type 2 diabetes; TCA: tricarboxylic acid cycle; TG: triglyceride; TMAO: trimethylamine N-oxide; TNF-α: tumor necrosis factor-alpha; UHPLC: ultra-high-performance liquid chromatography. ↑: increase; ↓: decrease.
